# Theta but not beta activity is modulated by freedom of choice during action selection

**DOI:** 10.1038/s41598-022-13318-7

**Published:** 2022-06-01

**Authors:** Emeline Pierrieau, Sarah Kessouri, Jean-François Lepage, Pierre-Michel Bernier

**Affiliations:** 1 Programme de Physiologie, Faculté de Médecine et des Sciences de la Santé, Université de Sherbrooke, Sherbrooke, Québec J1H 5N4 Canada; 2 Département de Pédiatrie, Faculté de Médecine et des Sciences de la Santé, Université de Sherbrooke, Sherbrooke, Québec J1H 5N4 Canada; 3 Département de Kinanthropologie, Faculté des Sciences de l’Activité Physique, Université de Sherbrooke, Sherbrooke, Québec J1K 2R1 Canada

**Keywords:** Decision, Decision, Neural circuits

## Abstract

Large-scale neurophysiological markers of action competition have been almost exclusively investigated in the context of instructed choices, hence it remains unclear whether these markers also apply to free choices. This study aimed to compare the specific brain dynamics underlying instructed and free decisions. Electroencephalography (EEG) was recorded while 31 participants performed a target selection task; the choice relied either on stimulus–response mappings (instructed) or on participants’ preferences (free). Choice difficulty was increased by introducing distractors in the informative stimulus in instructed choices, and by presenting targets with similar motor costs in free choices. Results revealed that increased decision difficulty was associated with higher reaction times (RTs) in instructed choices and greater choice uncertainty in free choices. Midfrontal EEG theta (4–8 Hz) power increased with difficulty in instructed choices, but not in free choices. Although sensorimotor beta (15–30 Hz) power was correlated with RTs, it was not significantly influenced by choice context or difficulty. These results suggest that midfrontal theta power may specifically increase with difficulty in externally-driven choices, whereas sensorimotor beta power may be predictive of RTs in both externally- and internally-driven choices.

## Introduction

At the neurophysiological level, making a decision is conceived as a competition between neuronal populations, each encoding a given option^1,2^. However, how and when this competition process occurs in the brain remains unclear^3^. Current decision-making models propose that competition proceeds in a distributed manner in the brain, through activation and inhibition of different brain circuits^4,5^. Electro- and magneto-encephalography (EEG and MEG respectively) have been used to investigate large-scale brain activity patterns of action competition in terms of oscillatory power, which is considered to reflect the degree of synchronization of underlying neuronal populations^6,7^. Using these tools, theta (4–8 Hz) power over the midfrontal cortex was identified as a potential marker of action competition in the context of cognitive conflict^8,9^, that is when the correct action among several options is harder to select based on external stimuli. Midfrontal theta activity has been associated with the activation of the underlying midfrontal region^10,11^, and with the increase in reaction time (RT) and variability in the selected option due to conflict^12^, potentially reflecting a sustained competition process between action representations for decision-making. Alternatively, beta (15–30 Hz) power over the sensorimotor cortex has also been associated with action competition during decision-making^13,14^, including cognitive conflict tasks^15^. Precisely, Donner et al. (2009) showed that lateralized beta-band activity is predictive of hand choices^13^, results that have been further replicated by Fischer et al. (2018) who demonstrated that beta-band activity can actually be used as a proxy of evidence accumulation and response thresholds in a flanker task^14^. Grent-‘t-Jong et al. (2013) showed that conflict in a flanker task was associated with lower beta power (i.e. increased beta desynchronization) over the contralateral sensorimotor cortex in a unimanual task^15^. Furthermore, the reduced power of sensorimotor beta activity appears predictive of RT^14,16^ and sensitive to action value^17,18^. This desynchronization of sensorimotor beta activity is thought to reflect a changing state of motor regions allowing the encoding of new motor commands^19^ and might be linked to the endogenous activation of action representations^20^.

Considering the evidence reviewed above, we expected midfrontal theta power to significantly increase (higher theta synchronization) and sensorimotor beta power to significantly decrease (higher beta desynchronization) when increasing choice difficulty, and thus action competition. However, to date, midfrontal theta and sensorimotor beta activities have only been associated with action competition in the context of instructed (stimulus-based) decisions. Indeed, studies investigating the specific patterns of brain oscillations modulated by action competition in free (action-based) choices are lacking. Neuroimaging studies have demonstrated an involvement of partly distinct brain regions^21,22^ and patterns of brain oscillations^23–25^ in instructed and free decisions, but whether these activities are specific to action competition is unclear. Therefore, the present protocol aimed to manipulate difficulty in both instructed and free choices. The latter was achieved by modulating choice uncertainty (i.e., participants’ preferences). Indeed, the influence of decision uncertainty on its difficulty has been well defined^69–73^. This was achieved by finding the point of subjective equality (PSE), that is the configuration in which the two presented options are chosen equally often, and thus the uncertainty maximal. Participants’ preferences were mainly based on expected motor costs. Previous studies using a similar manipulation of decision difficulty in tasks involving free hand choices showed modulations in RTs with choice uncertainty similar to what has been observed in perceptual decision-making tasks^26,27^.

Consequently, the objective of the present study was to determine if midfrontal theta power and sensorimotor beta power are specifically related to action competition in the context of instructed choices, or if their involvement extends to free choices. To achieve this aim, we compared the specific EEG activity associated with action competition in the two contexts of choice by comparing conditions of “difficult” vs “easy” choices (resulting in changes in relative preferences or RTs), while keeping similar sensory inputs, motor outputs, and differences in RTs between contexts.

## Results

**Figure 1:**
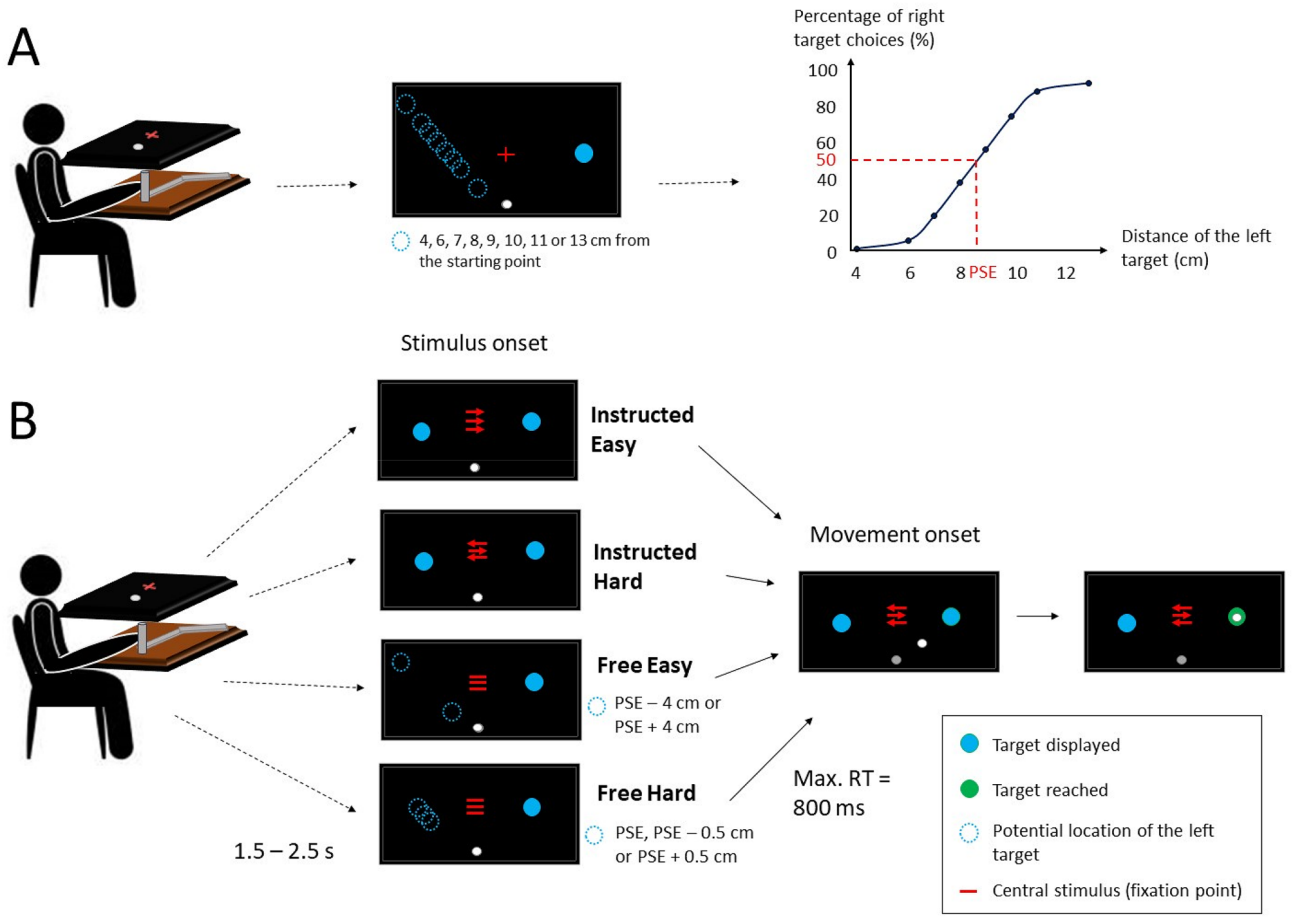
Experimental design. (**A**) Preliminary test. On the left, the scheme represents participants’ placement and the manipulandum used. Participants were asked to grasp a handle (gray cylinder) located below the screen with their right hand, which position was displayed on the screen in real time with a cursor (white dot). Black rectangles are schematic representations of the screen displayed in front of participants. The trial started with the appearance of a red fixation cross and the starting point (gray dot). Once participants placed their cursor into the starting point for 1.5 to 2.5 s, two targets appeared on the screen. The location of the left target varied across trials (all possible locations are represented with the dotted blue circles). The percentage of right target choices as a function of left target locations was fitted with a psychometric function to extract the PSE (i.e., left target location corresponding to the 50th percentile of right target choice). (**B**) Test. Participants’ placement was similar to the one used in the preliminary test. Trial timeline was also similar, except that at target onset the red fixation cross turned into a stimulus indicating the choice context (arrows: instructed, lines: free) and the direction of the target to reach in the case of an instructed choice (direction indicated by the central arrow). Movement onset was considered as the first time point at which the position of the cursor was located outside of the starting point. If the cursor ended inside of one of the two presented targets in free choices or inside of the target indicated by the central arrow in the case of instructed choices, the selected target turned green.

Participants (n = 31) performed a target selection task, in which they chose one of two visual targets presented on a screen. In addition to the targets, a central stimulus composed of either three arrows or three horizontal lines was also presented. Arrows informed of an instructed choice (Instructed) in which participants were asked to reach the target indicated by the direction of the central arrow. The direction of the two peripheral arrows could be congruent or not with the direction of the central arrow, hence modifying choice difficulty (congruent arrows: Instructed Easy; incongruent arrows: Instructed Hard; Fig. 1). Horizontal lines indicated a free choice (Free), meaning that participants were free to choose the target they preferred. Choice difficulty was manipulated by varying the distance of the left target from the starting point, thus modifying participants’ preferences (left target far from the point of subjective equality (PSE): Free Easy; left target close to the PSE: Free Hard; see Fig. 1 and Methods for details). Consequently, the effects of Context (Instructed or Free) and Difficulty (Easy or Hard) were tested factorially in the following behavioral and EEG analyses. Reaction times (RTs) were defined as the time separating stimulus onset from movement onset. If RTs exceeded 800 ms, the trial was aborted and an error message encouraging participants to initiate their movement faster was displayed on the screen. Spectral power was extracted from stimulus- and movement-locked epochs for theta and beta-band activity respectively, and was baseline-corrected based on the power recorded during a 500-ms window during the inter-trial interval preceding stimulus onset (Figures 3A, 5A; see “Methods” for details).

### Behavior

**Figure 2:**
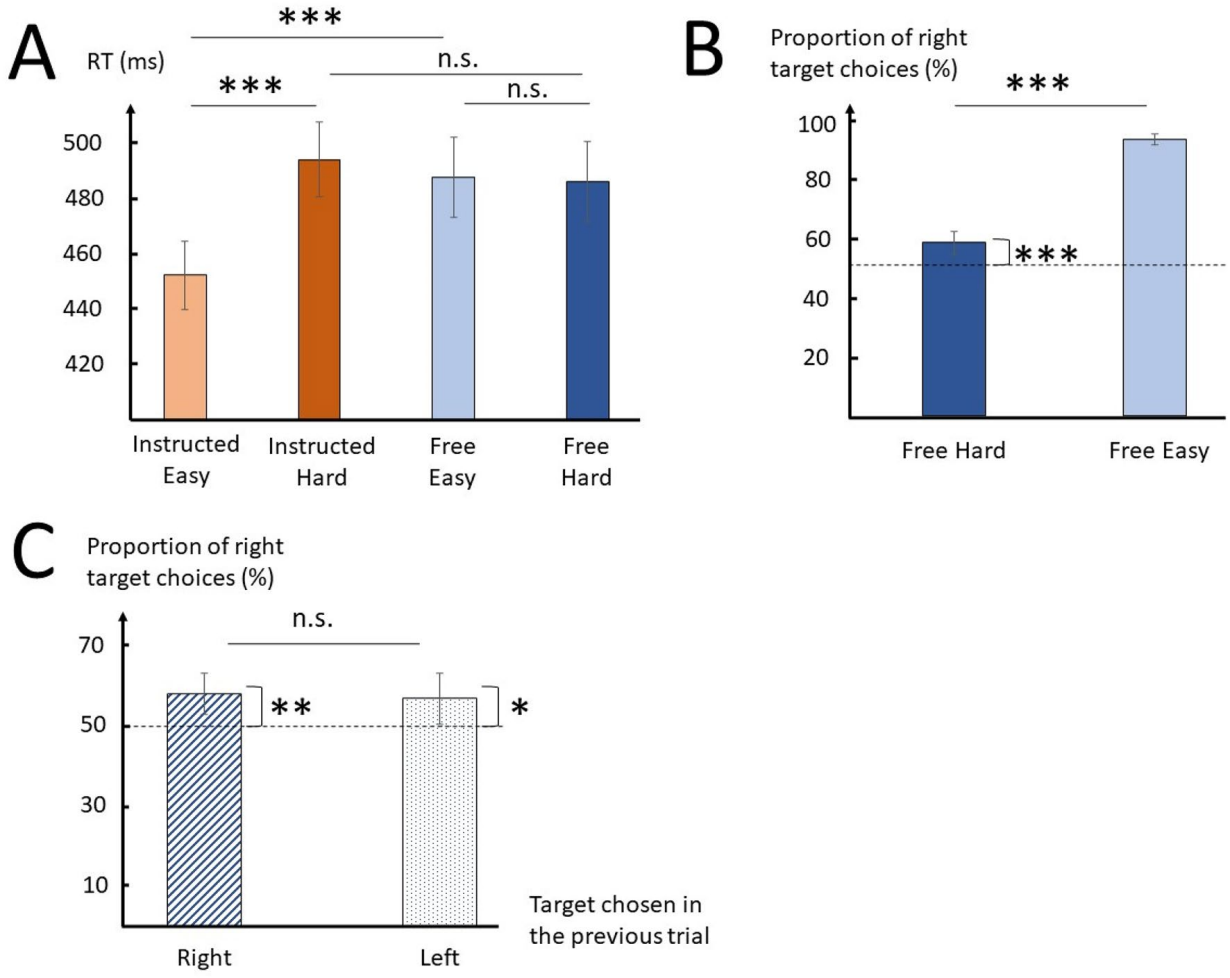
Effects of context and difficulty on RTs and target choices. (**A**) Average RT per condition. (**B**) Proportion of right target choices in Free, according to Difficulty. The dotted line indicates 50% of right target choices or PSE (right target chosen equally often than left). (**C**) Proportion of right target choices in Free Hard trials according to the target chosen in the immediately preceding trial (hatched bar = right target chosen, dotted bar = left target chosen). n.s. = non-significant p-value (p > 0.05), *p < 0.05, **p < 0.01, ***p < 0.001.

The analysis of reaction times (RTs) showed significant effects of Context (F(1,29) = 20.8, p = 10^–4^, η^2^p = 0.42), Difficulty (F(1,29) = 135.7, p = 10^–12^, η^2^p = 0.82), and an interaction (F(1,29) = 100.2, p = 10^–10^, η^2^p = 0.78). Post-hoc analysis showed that RTs were significantly slower in Instructed Hard than in Instructed Easy (Instructed Easy: mean ± SD = 453 ± 36 ms; Instructed Hard: mean ± SD = 495 ± 39 ms; t(29) = 12.8, p = 10^–11^, d = 2.33) but there was no significant difference in RTs between Free Hard and Free Easy (Free Easy: mean ± SD = 483 ± 41 ms; Free Hard: mean ± SD = 486 ± 41 ms; t(29) = 0.7, p = 1.0, d = 0.12; Fig. 2A). RTs between Instructed Hard and Free Hard were not significantly different (t(29) = 1.8, p = 0.320, d = 0.33).

Although the absence of difference in RTs between Free Easy and Free Hard is puzzling and questions the efficacy of our experimental design in modulating Difficulty in Free, the data nonetheless revealed a robust difference in the proportion of choices as a function of Difficulty. Indeed, the right target was chosen much more often in Free Easy (mean_FE_ = 93.8 ± 5.4%) than in Free Hard (mean_FH_ = 57.8 ± 10.5%, t(29) = 17.6, p < 10^–16^, d = 3.21; Fig. 2B), arguing for a greater level of uncertainty in Free Hard. Still, the proportion of right target choices in Free Hard was significantly higher than 50% (t(29) = 4.0, p = 0.001, d = 0.72), suggesting the existence of a bias for the right target. This bias was observable irrespective of the previous choice: the percentage of right target choices was significantly higher than 50% when participants chose the right target in the previous trial (t(29) = 3.0, p = 0.006, d = 0.54), and when they chose the left target in the previous trial as well (t(29) = 2.1, p = 0.046, d = 0.38; Fig. 2C). There was no significant difference in the percentage of right target choices according to which target was previously chosen (t(29) = 0.2, p = 0.806, d = 0.05), suggesting that the previously chosen target did not significantly influence choices in Free Hard trials. Overall, the large difference in the proportion of right target choices between Free Hard and Free Easy trials, the absence of significant repetitiveness of choices regarding target choice in the previous trial, and the fact that all participants showed at least one of the PSE conditions comprised between 25 and 75% of right target choice (see “Methods”) suggest that the current paradigm successfully modulated choice uncertainty despite the absence of difference in RT between conditions. Additional analyses demonstrated that the absence of significant RT difference between Free Easy and Free Hard might be at least partly explained by modulations of RT due to left target distance in Free (Supplementary material, Figure S1).

### EEG

#### Theta activity (4–8 Hz)

**Figure 3:**
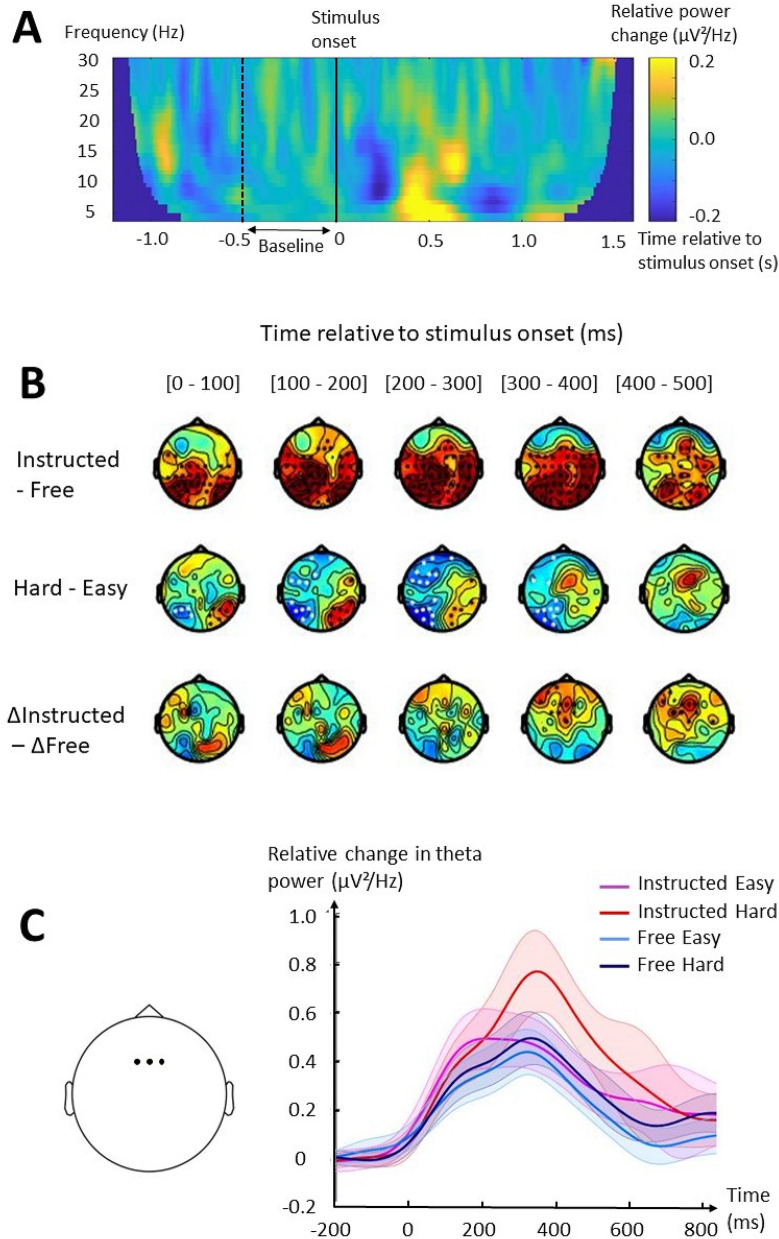
Fluctuations of theta power as a function of Context and Difficulty. (**A**) Time–frequency plot of the mean difference in spectral power between ΔInstructed and ΔFree at midfrontal electrodes (Fz, F1, F2). Data from the entire epoch is represented (−1.2 to 1.6 s around stimulus onset, indicated by the solid black line). This spectral power was computed as the relative change from a baseline period, corresponding to 0.5 to 0 s before stimulus onset (period separating the dotted and solid black lines). (**B**) Results of the cluster-based permutation tests for each paired comparison showing significant clusters. Each topographical plot represents the average difference in theta power between the tested conditions for each electrode in 100-ms windows, time-locked to stimulus onset. Hot colors indicate an increase and cold colors a decrease in theta power. Black dots indicate electrodes belonging to a significant positive cluster (theta increase) and white dots indicate electrodes belonging to a significant negative cluster (theta decrease). Δ refers to the difference associated with Difficulty (Hard–Easy). (**C**) On the left panel, black dots indicate the electrodes used as the midfrontal cluster (Fz, F1, F2). On the right panel, the plot shows the average baseline-normalized theta power at the midfrontal cluster as a function of time. Time is reported according to stimulus onset.

Cluster-based permutation tests revealed that theta power was significantly enhanced in Instructed as compared to Free, shown by a broad positive cluster (t_sum_ = 5051.5, p = 0.001) spanning parieto-occipital and midfrontal electrodes early and late in the RT period, respectively (Fig. 3B, top panel). Because there was an overall significant difference in RTs between Instructed and Free but not between Instructed Hard and Free Hard (see behavioral results above), we then compared Instructed Hard and Free Hard to verify that the cluster previously found was not a by-product of the RT difference between conditions. The analysis showed a significant positive cluster (t_sum_ = 6963.0, p = 0.001) over the same regions than in the previous analysis with a similar posterior-to-anterior pattern. Thus, Context significantly influenced theta activity during the RT period, specifically by a broad parieto-frontal increase in Instructed as compared to Free. Theta activity was then compared across Difficulty levels, pooled across Context. Theta activity was significantly reduced in Hard as compared to Easy condition in a negative cluster located in the left hemisphere, from occipito-parietal to frontal regions, 150 to 440 ms after stimulus onset (t_sum_ = −452.3, p = 0.005). The activity from a right occipito-parietal cluster was significantly enhanced in Hard condition 40 to 190 ms after stimulus onset (t_sum_ = 343.6, p = 0.011) (Fig. 3B, middle panel). Finally, we tested for an interaction between Context and Difficulty by comparing the difference due to Difficulty within each Context. We obtained similar results to the previous analysis, with a significant negative cluster 160 to 330 ms after stimulus onset (t_sum_ = −384.7, p = 0.009) located in left occipito-parietal and frontal electrodes, and a significant positive cluster 40 to 200 ms after stimulus onset (t_sum_ = 355.9, p = 0.009) located in right occipito-parietal electrodes.

Because the Difficulty-induced RT increase was apparent only in Instructed (see behavioral results), we controlled for the significance of the previous result by comparing Instructed and Free after having introduced a significant difference in RTs in the latter condition. This was achieved by removing 25% of the longest RTs in Free Easy and 25% of the shortest RTs in Free Hard from the analysis. The rejection was done only if the number of trials in the condition exceeded 30. This procedure allowed to obtain a similar difference in RTs between Free Hard and Free Easy, and between Instructed Hard and Instructed Easy conditions (mean_INSTRUCTED_ = 42.0 ± 18.1 ms, mean_FREE_ = 41.2 ± 16.8 ms; t(29) = 0.18, p = 0.9, d = 0.03) while keeping enough trials to ensure a correct signal/noise ratio for the analysis of brain oscillations (Free Easy: 35 ± 2 trials, Free Hard: 40 ± 12 trials). This correction (referred to as RTcorFree in the following text) annulled the left and right parieto-frontal cluster found in the previous analysis, and revealed a significant late positive midfrontal cluster 420 to 500 ms after stimulus onset (t_sum_ = 212.8, p = 0.046) (Fig. 3B, bottom panel). These results suggest that the modulations of theta activity found in bilateral parieto-occipital electrodes were likely due to temporal shifts caused by the Difficulty-induced increase in RT observed in Instructed but not in Free, whereas the increase of midfrontal theta activity was specifically enhanced by Difficulty in Instructed as compared to Free. This result was confirmed by computing a general linear mixed model (GLMM) including midfrontal theta at late RT (300 to 500 ms after stimulus onset) as a dependent variable, Context and Difficulty as independent variables, RT as a covariate and participant as a random variable. It revealed significant effects of Context (F(1,940) = 12.7; p = 0.0004), Difficulty (F(1, 951) = 4.5; p = 0.035) and an interaction between the two factors (F(1,955) = 4.2; p = 0.042) but no significant effect of RTs (F(1,129) = 0.0; p = 0.861) on average midfrontal theta power.

The comparison between Instructed Hard and Instructed Easy showed several clusters detected in the previous analysis: a positive cluster 50 to 200 ms after stimulus onset (t_sum_ = 371.2, p = 0.013) located at right parieto-occipital electrodes, another positive cluster 300 to 500 ms after stimulus onset (t_sum_ = 570.7, p = 0.004) centered on midfrontal electrodes, and a negative cluster 170 to 330 ms after stimulus onset (t_sum_ = −226.7, p = 0.037) located in left parieto-occipital electrodes. In contrast, no significant cluster was found when comparing theta power in Free Hard and Free Easy, even with RTcorFree trials. Consistent with these results, the time course of midfrontal theta power showed a distinct peak in Instructed Hard 300 to 600 ms post-stimulus (Fig. 3C).

**Figure 4:**
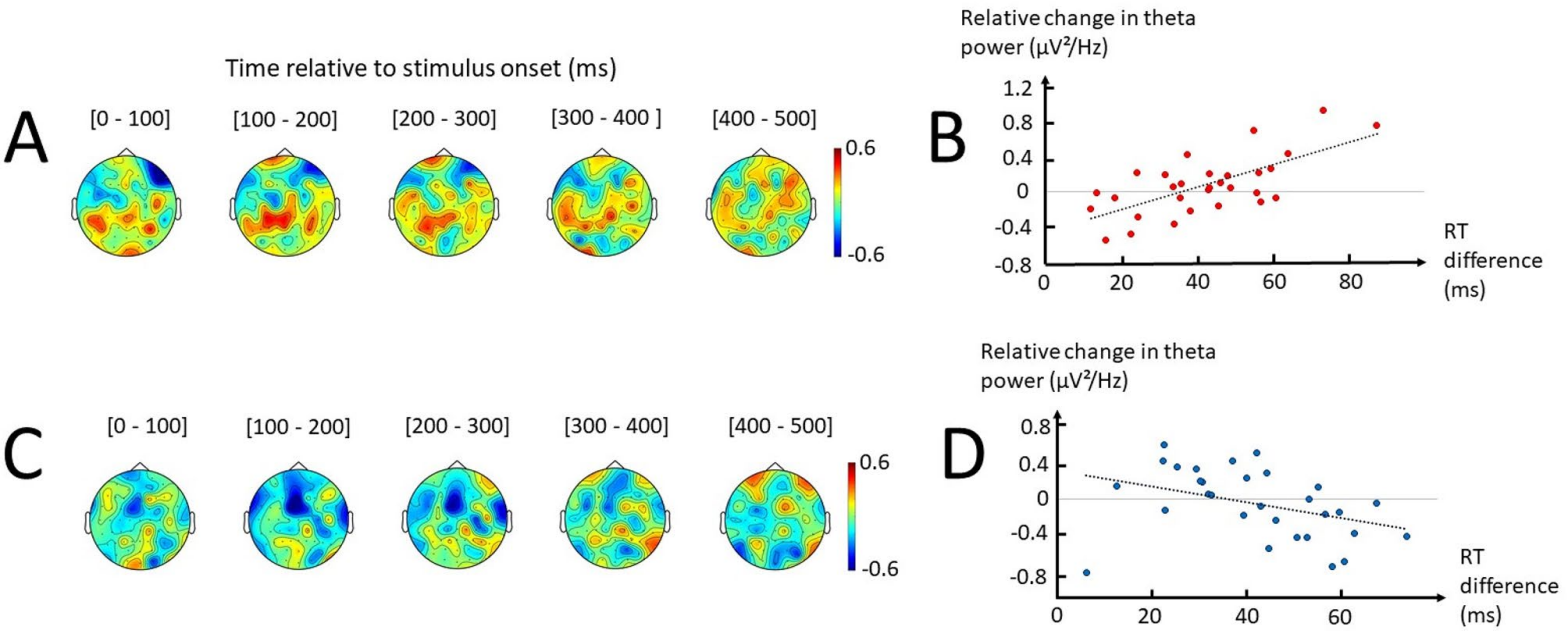
Correlations between theta power and RT difference associated with Difficulty in Instructed and Free. (**A**) Topographical representation of Spearman’s coefficients of the correlation between the difference in baseline-normalized theta power and RT in Instructed as a function of Difficulty (Instructed Hard–Instructed Easy) performed on each electrode separately. Each topographical plot represents the average Spearman’s coefficient in 100-ms windows defined according to target onset. Black dots indicate the electrodes with the highest absolute coefficient, specifically represented in **B**. (**B**) Linear fit between the difference in baseline-normalized theta power at parietal electrodes (CPz, CP1, CP3) and the difference in RT in Instructed as a function of Difficulty. (**C**) Topographical representation of Spearman’s coefficients of the correlation between the difference in baseline-normalized theta power and RT in Free as a function of Difficulty (Free Hard–Free Easy) performed on each electrode separately. Black dots indicate the electrodes with the highest absolute coefficient, specifically represented in **D**. (**D**) Linear fit between the difference in baseline-normalized theta power at midfrontal/left premotor electrodes (Fz, F1, FC1) and the difference in RT in Free as a function of Difficulty.

Finally, a correlation analysis including all electrodes revealed that the increase in RT between Instructed Hard and Instructed Easy correlated with an increase in theta power in left parietal regions, being maximal at CPz, CP1, CP3 (Spearman’s rho = 0.57, p = 0.001; Fig. 4A). This correlation remained significant when using a linear fit (Pearson’s r = 0.68, p < 0.001; Fig. 4B). Additionally, no significant correlation was found between the theta power in this left parietal cluster of electrodes (CPz, CP1, CP3) and RT difference between Free Hard and Free Easy using RTcorFree trials (Spearman’s rho = −0.23, p = 0.217; Pearson’s r = −0.26, p = 0.162). Nonetheless, in RTcorFree trials, the RT increase between Easy and Hard appeared best correlated with a decrease in theta power in a midfrontal/left premotor regions (Fz, F1, FC1) (Spearman’s rho = −0.51, p = 0.004; Fig. 4C), not better explained by a linear fit (Pearson’s r = −0.39, p = 0.036; Fig. 4D). Both correlations appeared maximal between 100 and 300 ms after target onset.

Overall, theta power was broadly increased in Instructed as compared to Free despite similar sensory inputs, motor outputs, and RTs. A late increase in midfrontal electrodes appeared specifically related to action competition in Instructed because it was found in Instructed Hard in comparison to Instructed Easy, and subsisted when equating RT differences between conditions. Furthermore, between-participants correlation analyses showed that in Instructed, the increase in RT associated with Difficulty was associated with greater theta synchronization in left parietal activity, whereas in Free, a similar increase in RT was related to lesser theta synchronization in midfrontal electrodes.

#### Beta activity (15–30 Hz)

**Figure 5:**
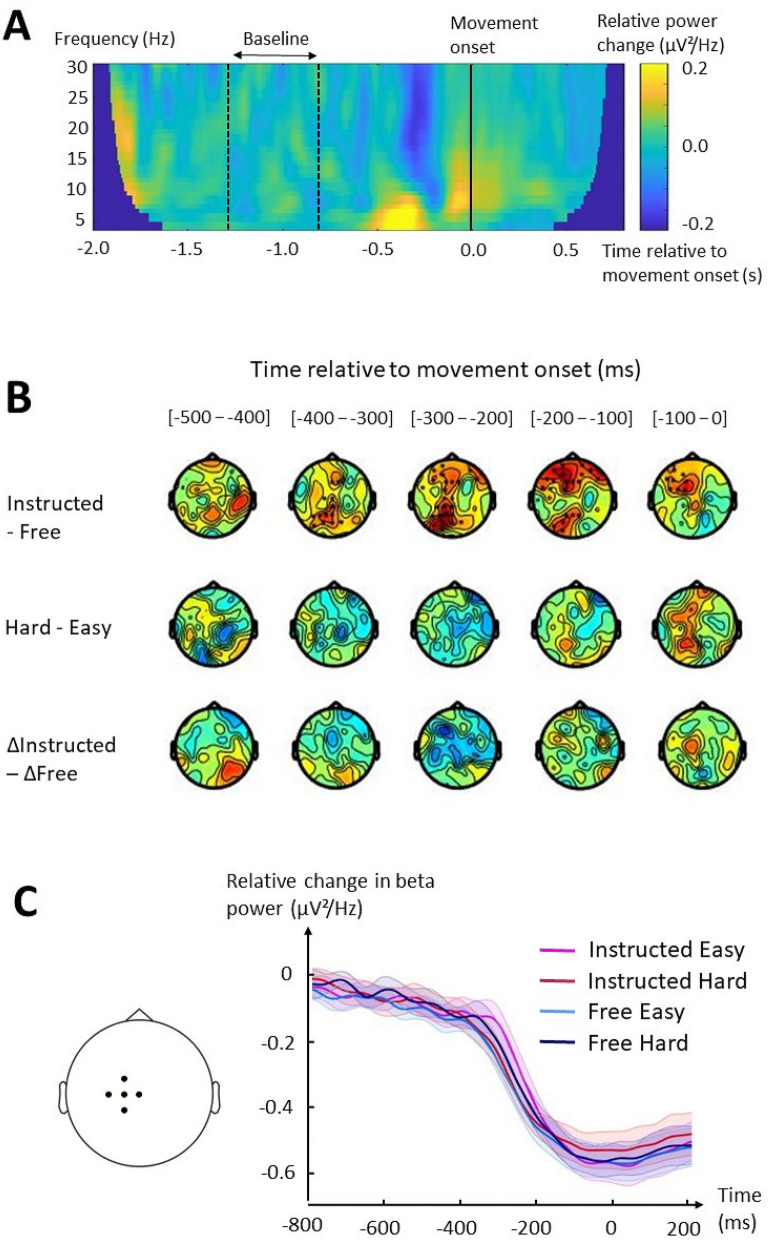
Fluctuations of beta power as a function of Context and Difficulty. (**A**) Time–frequency plot of the mean difference in spectral power between ΔInstructed and ΔFree at left sensorimotor electrodes (C1, FC1, C3, Cz, CP1). Data from the entire epoch is represented (−2.0 to 0.8 s around movement onset, indicated by the solid black line). This spectral power was computed as the relative change from a baseline period, corresponding to 1.3 to 0.8 s before movement onset (period separating the dotted black lines). (**B**) Results of the cluster-based permutation tests for each paired comparison. Each topographical plot represents the average difference in beta power between the tested conditions for each electrode in 100-ms windows, reported according to movement onset. Hot colors indicate an increase and cold colors a decrease in beta power. Black dots indicate electrodes belonging to a significant positive cluster (beta increase) and white dots indicate electrodes belonging to a significant negative cluster (beta decrease). Δ refers to the difference associated with Difficulty (Hard–Easy). (**C**) On the left panel, black dots indicate the electrodes used as the contralateral motor cluster (C1, C3, Cz, FC1, CP1). On the right panel, the plot shows average baseline-normalized beta power at this contralateral motor cluster as a function of time. Time is reported according to movement onset.

The comparison of beta power according to Context highlighted a significant cluster (t_sum_ = 953.8, p = 0.001) of left parieto-occipital and (pre)frontal electrodes 350 ms to 80 ms before movement onset, which showed higher beta power in Instructed than in Free (Fig. 5B, top panel). The comparison of beta power between Instructed Hard and Free Hard showed similar results: an early right parieto-occipital positive cluster 500 to 410 ms before movement onset (t_sum_ = 150.6, p = 0.038) and a late left prefrontal positive cluster 160 to 90 ms before movement onset (t_sum_ = 148.5, p = 0.040). No significant beta cluster appeared influenced by Difficulty (Fig. 5B, middle panel). The comparison of beta power as a function of Difficulty within Context (i.e., interaction) showed lower beta power in a broad left fronto-parietal cluster (t_sum_ = −538.5, p = 0.004) 340 to 210 ms before movement onset with Difficulty in Instructed as compared to Free. However, using RTcorFree trials annulled the significance of this cluster (Fig. 5B, bottom panel), suggesting that this modulation of beta power might have been related to the RT difference between conditions. Consistent with this hypothesis, a GLMM including sensorimotor beta power 400 to 200 ms before movement onset as a dependent variable, Context and Difficulty as independent variables, RT as a covariate and participant as a random variable showed no significant effects of Context (F(1,946) = 0.0; p = 0.906) and Difficulty (F(1, 955) = 1.3; p = 0.262) nor an interaction between the two factors (F(1,955) = 2.4; p = 0.120) but a significant influence of RTs (F(1,191) = 21.4; p = 10^–6^) on average sensorimotor beta power.

The comparison of beta power between Instructed Hard and Instructed Easy trials showed a broad negative cluster (t_sum_ = −474.7, p = 0.005) located in fronto-central electrodes 330 to 220 ms before movement onset, and a left central positive cluster (t_sum_ = 237.1, p = 0.023) occurring 120 to 0 ms before movement onset. As with theta power, no significant cluster was found when comparing beta power in Free Hard and Free Easy trials, even when using RTcorFree trials. Visual depiction of the time course of sensorimotor beta power did not show any noticeable modulation across conditions (Fig. 5C).

**Figure 6:**
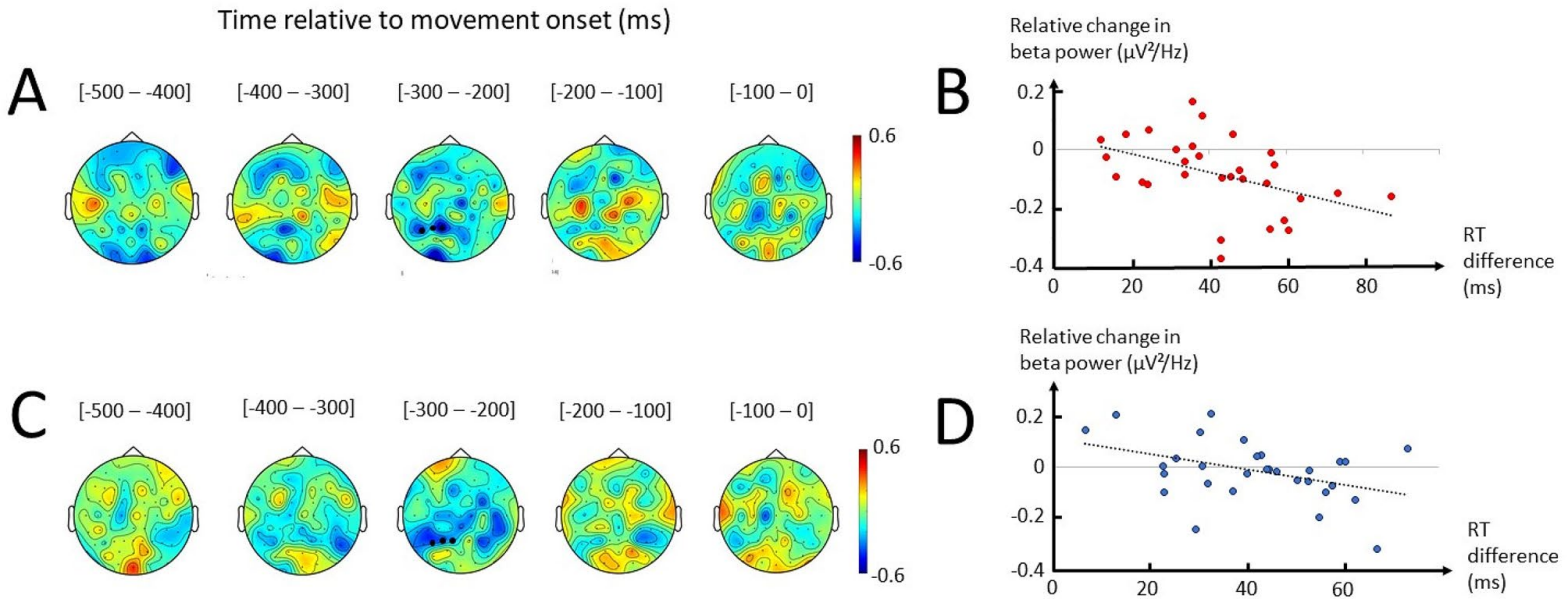
Correlations between beta power and RT difference associated with Difficulty in Instructed and Free. (**A**) Topographical representation of Spearman’s coefficients of correlation between the difference in baseline-normalized beta power and RT in Instructed as a function of Difficulty (Instructed Hard–Instructed Easy) performed on each electrode separately. Each topographical plot represents the average Spearman’s coefficient in 100-ms windows defined according to movement onset. Black dots indicate the electrodes with the highest absolute coefficient, specifically represented in **B**. (**B**) Linear fit between the difference in baseline-normalized beta power at left parietal electrodes (P1, P3, P5) and the difference in RT in Instructed as a function of Difficulty. (**C**) Topographical representation of Spearman’s coefficients of correlation between the difference in baseline-normalized beta power and RT in Free as a function of Difficulty (Free Hard–Free Easy) performed on each electrode separately. Black dots indicate the electrodes with the highest absolute coefficient, specifically represented in **D**. (**D**) Linear fit between the difference in baseline-normalized beta power at left parietal electrodes (P1, P3, P5) and the difference in RT in Free as a function of Difficulty.

Similar to theta, a correlation analysis was performed between the difference in beta and RT associated with Difficulty according to Context. A negative correlation between the difference in left parietal beta (P1, P3, P5) and RT emerged from the analysis in both Instructed and Free (using RTcorFree trials), 300 to 200 ms before movement onset (Fig. 6A, C). Correlation tests between the difference in beta power and RT observed at these left parietal electrodes (P1, P3, P5) revealed a significant negative correlation in Instructed (Pearson’s r = −0.45, p = 0.013; Spearman’s rho = −0.51, p = 0.004; Fig. 6B), slightly less significant but also noticeable in Free (Pearson’s r = −0.41, p = 0.023; Spearman’s rho = −0.33, p = 0.076; Fig. 6D).

Overall, sensorimotor beta power did not appear differently modulated by difficulty according to the choice context, and was negatively correlated with RTs in both choice contexts.

#### Control analysis with one-target trials

To further assess if the observed patterns of activity truly resulted from a decision process and not from different perceptual representations or motor planning across conditions, they were compared with one-target trials (1 T) oriented toward the same target than the one chosen in Instructed and Free trials used in previous analyses (i.e., the right target; see Methods for details). To ensure similar differences in RTs between comparisons of two-targets and one-target trials, only Instructed Hard and Free Hard were used (see behavioral results). Contrasting choice and 1 T trials revealed overall higher theta and lower beta power in the former. Cluster-based permutation tests demonstrated qualitatively different modulations of theta power but similar modulations of beta power across decision contexts (Supplementary material; Figure S2). This result was confirmed by an ANOVA including activity pattern (midfrontal theta and sensorimotor beta) and Context as factors. It showed significant effects of activity pattern (F(1,28) = 19.8; p = 10^–4^; η^2^p = 0.42), Context (F(1,28) = 7.6; p = 0.010; η^2^p = 0.21), and a significant interaction between the two factors (F(1,28) = 9.7; p = 0.004; η^2^p = 0.26). Post-hoc Bonferroni-corrected t-tests demonstrated that midfrontal theta power was significantly increased in Instructed as compared to Free (t(28) = 3.0; p = 0.011; d = 0.56) whereas sensorimotor beta power was not significantly modulated when comparing Instructed and Free (t(28) = −0.9; p = 0.796; d = −0.16). Therefore, while both midfrontal theta power and sensorimotor beta power were sensitive to the addition of another target (and thus potentially to action competition), only midfrontal theta power appeared significantly influenced by choice context.

## Discussion

The main finding of the present work is that the well-described increase in midfrontal theta power with action competition was specific to instructed choices, as it was not found in the context of free choices. Despite significant broad changes in beta power with choice context, these did not appear related to difficulty as they were not found in the interaction analysis comparing overall beta activity related to difficulty across choice contexts, nor in the control analysis comparing overall beta activity in choice trials to one-target trials. Additionally, a similar negative correlation between RT difference in easy and hard trials and beta power was found in instructed and free choices. Comparison with one-target trials further demonstrated that these activity patterns were specifically found in a decision context.

The increase in midfrontal theta power with difficulty in instructed decisions replicates results from several studies using comparable tasks^9,46,47^. Midfrontal theta has been proposed as an electrophysiological marker of cognitive control and conflict by reflecting the activation of underlying cortical regions^12,48^. Indeed, several studies using functional magnetic resonance imaging and intracerebral recordings have shown that midfrontal regions such as the anterior cingulate cortex and the supplementary motor area are involved in conflict processing^49–51^, and that activity within these regions generates oscillations in the theta-band^10,11^. However, a striking result from the present study is that this commonly observed midfrontal theta increase was not observed when increasing difficulty in free choices. This observation is consistent with the hypothesis that externally- and internally-driven decisions are resolved through at least partially distinct neural circuitry^52^. In that regard, it has been proposed that midfrontal regions are mainly involved in external goal selection, while sensorimotor regions would be rather involved in internal effector and movement selection^22^. In support of this view, the posterior and lateral parietal areas have been shown to be causally related to the decision process underlying free hand choices^26,53,54^. A more specific contribution of midfrontal regions in stimulus–response mappings^21^ and of frontoparietal regions such as the parietal and the premotor cortex in free choices^21,24,25^ have been suggested by previous studies comparing instructed and free choices with one effector. However, decision difficulty was not manipulated in these studies, hence whether there exists a regional specificity of the action competition process according to choice context remained unclear. The present study expands on these earlier findings by showing that selection might be computed by a distinct neural circuitry in instructed and free choices, with a lesser (or at least different) involvement of midfrontal regions in the latter. This suggests that the increase in midfrontal theta power may be associated with the computation of stimulus–response mappings and cognitive control^52^, but may not represent a global marker of action competition.

Although they encompassed different electrodes (left parietal in instructed choices, midfrontal in free choices), correlations between changes in theta power and RT difference between easy and hard trials were opposite according to the context of choice. Activation of midfrontal regions such as the supplementary motor area (SMA) has been associated with the deliberation process occurring in free choices based on self-paced movements^55^, which are noticeably different from the present task by the non-restricted deliberation time allowed to participants and the absence of an external stimulus. The involvement of SMA in instructed choices based on cognitive rules has also been observed^56–58^, but evidence for its role in free choices based on expected action costs is lacking. Additionally, the electrodes where the correlation was maximal were located slightly left of the midline. Even though the limited spatial resolution of scalp EEG does not allow to reliably determine the origin of this change in neuronal activity, one hypothesis consistent with the current literature on free choices would be an involvement of left premotor regions^21,23,59^. Whether or not the contralateral premotor cortex can host a competition between action representations is still actively debated^60–62^. Further studies investigating action competition in free choices using a single effector with equipment allowing for a better spatial resolution, such as functional magnetic resonance imaging (fMRI) or MEG recordings, will be needed to disentangle the role of midfrontal regions in this context.

Results revealed an absence of significant difference in sensorimotor beta desynchronization related to action competition before movement onset in instructed and free choices, and a similar negative correlation between modulations of left parietal beta power and RT difference associated with difficulty in both types of choices. This decrease in sensorimotor beta power is supposed to be associated with a decrease in local inhibition^19^, related to a higher encoding capacity to allow the formation of a new motor command^63^. Considering that only trials comprising movements toward the right target were kept for analysis, one could argue that the comparable sensorimotor beta desynchronization across conditions was due to the preparation of a similar motor output. However, several studies report a modulation of sensorimotor beta power due to decision uncertainty or increased difficulty^15,16,64,65^, suggesting that sensorimotor beta power might be related to action competition^64,66^. Consistent with these studies, our results showed a greater beta desynchronization in choice trials (similarly in instructed and free choices) as compared to one-target trials directed toward the same target. One hypothesis would be that sensorimotor beta power reflects action competition irrespective of the external or internal nature of decisional variables, consistent with the perspective of an automatic specification and competition process between action possibilities in sensorimotor regions^2^. Additionally, the cerebral substrates of the integration of action costs into the decision process are still unresolved^31,67,68^, such that whether or not a competition between their representations should be reflected in modulations of sensorimotor beta power remains uncertain.

Importantly, interpretations regarding the large-scale dynamics of a neural competition based on expected action costs are mitigated in the present study due to the absence of difference in RT between easy and hard trials in free choices. Given the negative correlation found between the increase in RT and beta power, it may partly explain why a significant cluster of sensorimotor beta desynchronization appeared only when increasing decision difficulty in instructed but not in free choices. It was an unexpected result given that the uncertainty of target choice appeared effectively modulated, which has been shown to influence participants’ RT in effector selection tasks^26,27^ as well as in decision-making models^69,70^. The absence of significant modulation of RT cannot be explained by the repetitiveness of choice, as participants’ choices were not biased toward the previously chosen target in free hard trials. Nonetheless, participants globally tended to choose the right target more often than the left one, suggesting that the use of a single spatial location for the right target over the entire experiment may have led to a default bias toward the right target. However, Cos et al. (2011) used a similar task to ours and also did not report significant differences in RT in spite of the use of several target configurations^29^, suggesting that it may be related to other variables. One possibility highlighted by Figure S1 is that the distance of the left target influenced RTs and masked the difference in RT due to uncertainty between easy and hard trials. Indeed, in the free easy condition the left target was located farther from the starting point than in the free hard condition (PSE + 4 cm and PSE respectively), which may have increased RTs in the free easy condition. An effect of target distance on RTs is further supported by the significantly lower choice RTs when the left target was located at PSE—4 cm than when it was either at PSE or PSE + 4 cm, in addition to the significantly longer simple RTs for trials oriented toward PSE + 4 cm as compared to PSE and PSE − 4 cm. Overall, these results suggest that in the present task RTs might have only partially reflected action competition. An interesting future research avenue would be to investigate the specificity of brain activity related to action competition using a task that modulates the perceived effort while keeping target distance as well as other movement parameters that could influence RT constant between conditions.

In conclusion, midfrontal theta synchronization appeared as a specific marker of action competition in instructed choices in our task, whereas sensorimotor beta desynchronization was associated with action competition in instructed and free choices. Correlations between changes in theta power and RT with difficulty were opposite according to the context of choice, while a negative correlation between changes in beta power and RT with decision difficulty was observed in both contexts. Together these results highlight partly distinct activity patterns underlying action competition in instructed and free choices, thus demonstrating different sensitivities of brain rhythms to the choice context and helping disentangle the functional role of these activity patterns in the decision process.

## Methods

### Participants

Thirty-one (31) participants (15 females, 25.9 ± 4.5 (mean ± SD) years old) were recruited for this study. All participants had a normal or corrected-to-normal vision. All were right-handed based on self-report and were free of any known neurologic or psychiatric condition. Participants gave their informed written consent and received a $20 CAD compensation. All procedures were approved by the University of Sherbrooke institutional review board and ethics committee. The experiment conformed to the standards set by the 1964 Declaration of Helsinki.

### Experimental task

#### Setup

The experimental setup consisted of a table supporting a 20-inch computer monitor that projected visual stimuli onto a mirror positioned horizontally in front of the participants. The monitor (Dell P1130 20-inch monitor; resolution: 1024 × 768; refresh rate: 150 Hz) was mounted face down 29 cm above the mirror with the latter positioned 29 cm above the table surface. Participants' movements were recorded with an acquisition frequency of 100 Hz, using a two-joint manipulandum composed of two lightweight metal rods with a potentiometer at the hinges of the manipulandum. Participants performed their movements by grasping a handle located at the mobile end of the manipulandum with their right hand and sliding it over the table. The position of the handle (and thus participants’ hand) was kept visible for the participants using a cursor projected on the monitor. This system provided constant visual feedback of participants’ hand position, like a computer mouse. A 64-electrodes actiCAP (extended 10/20 system, Brain Products) was positioned on participants’ head to record scalp EEG. This was done by first measuring the head dimensions in the sagittal and frontal planes to localize the vertex and positioned the reference electrode (FCz) over it. The EEG data were acquired using the BrainVision Recorder software 2.0 (Brain Products) with a sampling rate of 500 Hz. This setup has been used in previously published work^18,27^.

#### Overview

Participants were seated in front of the table and asked to reach visual targets (cyan-filled circles, diameter = 3 cm) with their right hand (see Fig. 1 for an illustration of the experimental design). Visual stimuli were presented using Psychtoolbox on MATLAB (MathWorks). Trials were initiated by placing the cursor (white filled circle, diameter = 0.6 cm) on a starting point (light gray filled circle, diameter = 0.6 cm) located at the center of the screen. Participants were told to place their chin on a small support, to keep their right arm in contact with the surface of the table, and to minimize postural changes during the experiment. Targets were presented on the right side (60°, right target) and on the left side (150°, left target) of the screen, corresponding to directions associated with low and high biomechanical costs, respectively^28^. Indeed, the biomechanical cost associated with a reaching movement can be represented as an ellipse of mobility, with a major axis corresponding to the lowest cost, most chosen directions, and a minor axis corresponding to the highest cost, least chosen directions^29,30^. The right target was kept at the same distance from the starting point throughout trials (10 cm), whereas the distance of the left target varied to manipulate the difference in motor costs between movements (see below). The order of presentation of the trials was varied pseudo-randomly so that the same configuration of targets (or experimental condition) was not presented twice consecutively to prevent repetitiveness of choices.

Trials started with the display of a red fixation cross 2 cm above the starting point on the screen. Once the cursor was placed on the starting point, participants were required to keep their gaze on the fixation cross for the entire trial duration. They were also asked to minimize eye blinks until they reached a target to avoid eye artifacts on the EEG signal during RT. After a variable delay (1.5 to 2.5 s), targets appeared on the screen. If participants did not initiate their movement 800 ms after stimulus onset, the trial was aborted and an error message encouraging participants to initiate their movement faster was displayed on the screen. The interest was to avoid trials with abnormally long RTs and to keep participants engaged in the task. Movements were required to end inside of the chosen target to ensure accuracy and thus comparability of trials across conditions. The experiment was divided into 3 phases described below.

#### Preliminary test

This test was performed in the first part of the experiment. This test aimed to determine the point of subjective equality (PSE) between the two presented targets. The PSE consisted in the virtual point corresponding to the left target distance for which the left and right targets had an equal probability of being selected (50%), based on studies evaluating choices between effectors^26,27^. Because we were interested in removing effector-specific activity in this study, we aimed at estimating the PSE between targets instead of effectors. To do so, the left and right targets were presented at each trial with the distance of the left target varying across trials (4, 6, 7, 8, 9, 10, 11, and 13 cm from the starting point) to manipulate the difference in motor costs between targets. Indeed, for the same distance between targets, participants’ choices have been shown to be strongly biased toward the low-cost right target^29–31^, and this preference appeared sensitive to target distance^29^. Participants performed 40 familiarization trials to get used to the task, and then 25 trials for each distance of the left target (8 × 25 = 200 trials in total). They were told to choose the target they prefer by paying attention to the position of targets and avoiding cognitive strategies such as always reach to the same target or alternating between targets. The proportion of left target choices for each distance of the left target was fitted into a sigmoidal curve using a psychometric function (FitPsycheCurveLogit in MATLAB) in order to determine the PSE.

#### Main test

The main test aimed to induce instructed (Instructed) and free (Free) decision contexts and to manipulate the level of difficulty in each context. Eriksen’s flanker task^32^ was used to study the activity specific to Instructed decisions. To do so, the fixation cross transformed into a visual stimulus composed of 3 red horizontal arrows at target onset. Participants were asked to reach the target indicated by the direction of the central arrow, which could be congruent or not with the direction of the peripheral arrows. This task has been widely used to investigate the neurophysiological correlates of action competition^33–35^ which is supposed to be sustained when presenting incongruent arrows. Therefore, congruent arrows formed the Instructed easy condition (Instructed Easy) and incongruent arrows the Instructed difficult condition (Instructed Hard). In Instructed trials, the left target was located at a distance corresponding to the PSE, pre-determined in the previous phase. In Free trials, the fixation cross turned into 3 red horizontal lines at target onset. Participants were told that this stimulus indicated that they were free to choose any of the presented targets. In Free context, the left target was located either at PSE − 4 cm or PSE + 4 cm, corresponding to the Free easy condition (Free Easy) because of the eccentric position of the left target strongly biasing the choice toward one of the targets (left target at PSE − 4 cm, right target at PSE + 4 cm), either at PSE − 0.5 cm, PSE or PSE + 0.5 cm, corresponding to the Free difficult condition (Free Hard) because of the uncertainty of choice generated by the position of the left target. Participants first performed 45 familiarization trials, then 180 Instructed trials (50 Instructed Easy directed to the right target, 40 Instructed Easy directed to the left target, 50 Instructed Hard directed to the right target and 40 Instructed Hard directed to the left target) interspersed with 210 Free trials (40 at PSE − 4 cm, 50 trials at PSE + 4 cm, 40 trials at PSE − 0.5 cm, 40 trials at PSE, 40 trials at PSE + 0.5 cm).

#### Control test

Trials consisted of the presentation of one target instead of two, to study the activity associated with sensory processing and motor planning without the decision process occurring in the previous tests. In the first 80 trials (20 × 4 target positions), participants were asked to reach the presented target. The latter was presented at the position of the right target or left target at eccentric positions (PSE − 4 cm, PSE + 4 cm) or PSE. In the last 80 trials, the targets were presented at the same positions but participants were required not to make any movement. The target appeared on the screen for 1 s. The interest was to extract the activity specific to the visual processing of the target, without the motor component. However, this last set of trials was not used in the present study.

### Data analysis

#### Behavior

Hand position was estimated in real-time with the coordinates of the cursor recorded with the two potentiometers located on the manipulandum. Movement onset corresponded to the first time point when the coordinates of the cursor were outside the starting point. RTs were calculated as the latency separating target appearance and movement onset. Trials with RTs exceeding 800 ms were removed from the analysis because as mentioned in the previous section, participants could not initiate a movement after this delay. To further ensure the representativity of the activity analyzed during RT, RTs below and above three standard deviations from the median were eliminated per condition to remove outliers. Trials in which movement velocity fell below one pixel per second outside of the presented targets were considered as missed-target trials and were also removed from the analysis. Errors in Instructed trials (i.e., the chosen target was not the one indicated by the central arrow) were also removed. To ensure the similarity of all trials in terms of motor outputs and to avoid differences in brain activity due to motor planning, only trials with movement directed toward the right target were kept for the EEG analysis. Consequently, only Instructed trials with a central arrow directed to the right target, and Free trials with a high preference for the right target (PSE + 4 cm) or equiprobable choice between targets (PSE − 0.5 cm, PSE, PSE + 0.5 cm) with the right target chosen were used for EEG analyses. In Free choices, because the aim was to manipulate difficulty by varying the preference for each target, an important stake was to make sure that all tested participants showed different choice probabilities according to the position of the left target. Only one participant showed a percentage of right target choice under 75% at PSE + 4 cm (51%) and was thus excluded from the analysis. All other participants showed a marked preference for the right target at PSE + 4 cm (median = 94%, range = [82%, 100%]). In Free Hard, target positions with a percentage of right target choice below 25% and above 75% were removed. All participants showed at least one target position with a percentage of right target choice comprised in this interval. Following all rejections, there remained for analysis an average of 49.9 trials (± 0.4) in the Instructed Easy, 49.7 trials (± 1.1) in the Instructed Hard, 44.6 trials (± 3.5) in the Free Easy, and 53.1 trials (± 18.3) in the Free Hard.

#### EEG

All EEG data were analyzed offline using custom MATLAB codes and functions from EEGLAB^36^ and Fieldtrip^37^. First, a bandpass filter between 1 and 125 Hz was applied on raw EEG signal, with a 59–61 Hz Notch filter to attenuate electrical noise. The signal was re-referenced to the average scalp potential. The data was then segmented into epochs of 2.8 s duration locked around target onset (−1200 to + 1600 ms) and movement onset (−2000 to 800 ms). This period was chosen to study brain activity during RT (0 to 800 ms maximum after target onset and before movement onset) with a 500-ms baseline preceding RT while keeping an interval long enough for the signal to be free of edge artifacts (lowest frequency of interest = 4 Hz, corresponding to edge artifacts of 750 ms [3*250 ms]) and ensuring no overlap between epochs for the following analysis. Independent component analysis (ICA) was applied to EEG data using the *runica* algorithm from the EEGLAB toolbox, in order to remove artifactual EEG activity associated with eye and head movements and other sources of noise^38^. A surface Laplacian transform was applied on the EEG data with artifactual components removed, using the erplab plugin from EEGLAB. The interest was to improve the spatial resolution by reducing the contribution of distant sources to the EEG signal^39,40^. Trials marked in the behavioral-based rejection were then removed, and the EEG signal was downsampled to 250 Hz to reduce computation time for time–frequency decomposition. The latter was performed afterward, using Morlet wavelets (4–80 Hz with 1 Hz steps). The wavelet cycles were increased at each frequency in 0.1 steps (starting from 3 to 10.6 cycles) to ensure a balance between sufficient temporal resolution at lower frequencies and frequency resolution at higher frequencies. Finally, the analyzed data were normalized for each condition by measuring the relative change from a 500-ms baseline preceding RT (−500 to 0 ms for data time-locked to target onset, −1300 to −800 ms for data time-locked to movement onset).

### Experimental design and statistical analysis

Data were compared regarding Context (Instructed or Free) and Difficulty (Easy or Hard) of choice. A 2 × 2 ANOVA (Context x Difficulty) was performed on RTs. Difficulty was also assessed by comparing the proportion of right target choices in Free Easy and Free Hard. The proportion of right target choices was also computed separately according to the previously chosen target in Free Hard, to test for a significant influence of the preceding choice. Bonferroni-corrected t-tests were used as post hoc analysis. Cohen’s d is reported to estimate effect size for t-tests and partial eta squared (η^2^p) for ANOVAs.

Time–frequency data were first analyzed with cluster-based permutation tests using the Fieldtrip toolbox^37^ on MATLAB. This method was used to identify clusters of electrodes showing statistically significant differences across conditions in a 500 ms period of interest following stimulus onset for theta power [4–8 Hz] and preceding movement onset for beta power [15–30 Hz]. Stimulus-locked data were used for the analysis of theta power and movement-locked data for the analysis of beta power because theta power is associated with stimulus processing^41–43^, whereas beta power is linked to movement preparation^44^. Monte Carlo permutations (n = 1000) were used to determine p-values for each cluster. A cluster-level correction was set to control for multiple comparisons, using the sum of t-values^45^. A cluster was defined as at least two neighboring electrodes (located at less than 4 cm from each other) showing statistically significant t-values. The five following contrasts were performed with cluster-based permutation tests: Instructed vs Free, Easy vs Hard, Instructed Hard–Instructed Easy (Δ Instructed) vs Free Hard–Free Easy (ΔFree), Instructed Hard vs Instructed Easy and Free Hard vs Free Easy. Given that RTs did not appear to reliably reflect decision difficulty in the present task (see Results section for details), general linear mixed models (GLMMs) including Context (Instructed or Free) and Difficulty (Easy or Hard) as independent variables, RTs as a covariate and participants as a random variable were performed on midfrontal theta power and sensorimotor beta power separately and jointly (i.e., by adding activity patterns in the model as an independent variable).

In addition, global correlation tests (Spearman’s rho) between differences in power values and RTs as a function of Difficulty were conducted in each choice context and electrode per 100-ms windows (over the 500-ms period of interest). The aim of this analysis was to determine whether modulations in theta and beta power with RT significantly changed across choice context.

Finally, a control analysis was performed using cluster-based permutation tests to compare Instructed Hard and Free Hard with one-target trials. The goal was to assess the specificity of the findings obtained in previous analyses to the decision process, by subtracting activity due to the perception and motor preparation toward the selected target. Only one-target trials directed toward the right target were used. Results were deepened by conducting an ANOVA with Context and activity pattern (midfrontal theta or sensorimotor beta) as factors.

Apart from the cluster-based permutation tests, all the statistical tests were computed using Jamovi v. 1.2.27 (the jamovi project, 2019, computer software, retrieved from https://www.jamovi.org), a software that implements R statistical language (R Core Team, 2018, R: a language and environment for statistical computing, computer software, retrieved from https://www.cran.r-project.org/).

## Supplementary Information

Supplementary Information.

## Data Availability

The datasets analyzed in the present study are available from the corresponding author upon reasonable request.
