# Supplementary material for: Theta but not beta activity is modulated by freedom of choice during action selection
Source: Sci Rep. 2022 Jun 1;12:9115. doi: 10.1038/s41598-022-13318-7 (PMC9160249; doi:10.1038/s41598-022-13318-7)
Supplement: Supplementary file 1 — Supplementary Information. [file 41598_2022_13318_MOESM1_ESM.docx]

Figure S1


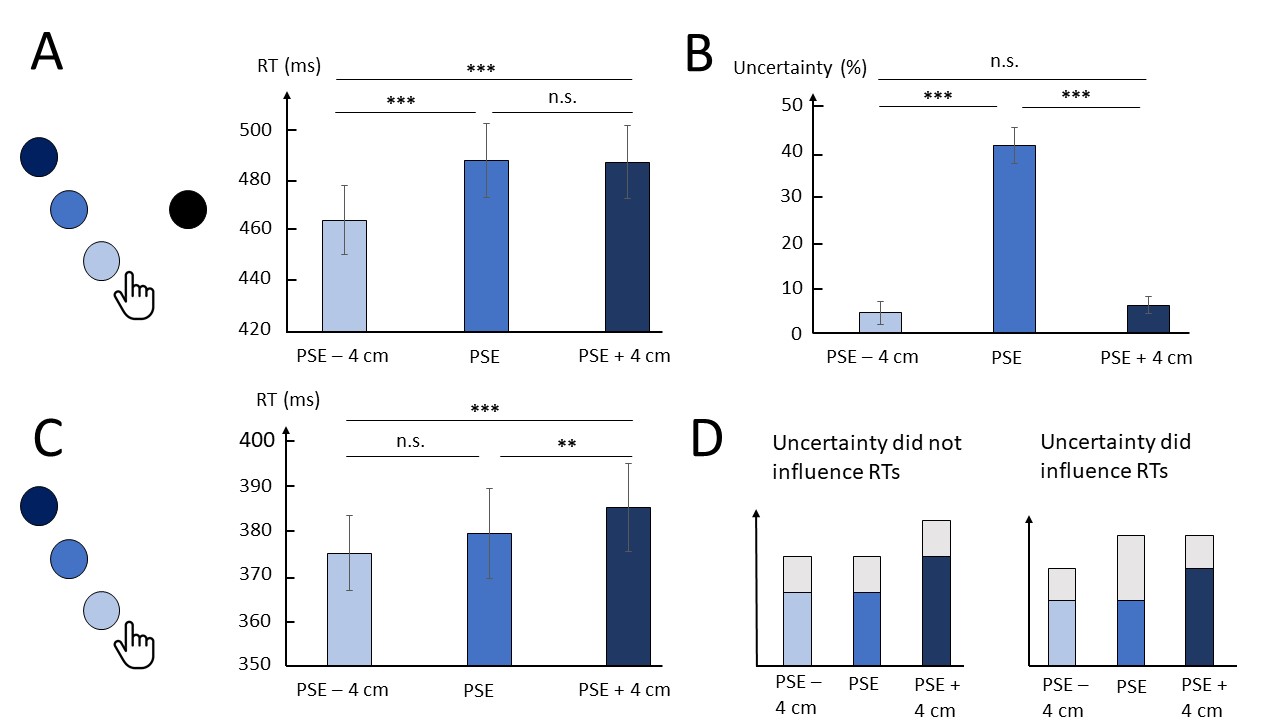


**Effect of left target distance on reaction times and uncertainty**. A. RTs according to left target distance in two-target trials (Free). B. Difference from 0 in the mean proportion of choice toward the least preferred target in each condition (PSE – 4 cm: right target; PSE and PSE + 4 cm: left target) as a quantification of uncertainty (the higher the difference, the more uncertain the choice). C. RTs according to left target distance in one-target trials. D. Expected pattern of results for RTs in Free (as represented in A) if choices were not influenced by uncertainty (left panel) and if they were (right panel). Blue areas indicate the portion of RTs explained by sensorimotor delays needed for motor planning toward each target location (RTs from one-target trials, see C) and gray areas represent the added delay due to choice. As represented in the right panel, this choice-related delay should be longer for PSE than for the other two conditions if uncertainty significantly influenced RTs, given that uncertainty was significantly higher in this PSE condition (see B). Note that the pattern of results supported by the hypothesis of a significant influence of uncertainty on RTs (right panel) resembles the one actually found (see A).

Detailed analysis:

No significant difference was found when comparing RTs between PSE and PSE + 4 cm trials (Bonferroni-corrected t-tests; t(29) = 0.3; p = 0.792; d = 0.05; mean RT_PSE_ ± SD = 489 ± 41 ms; mean RT_PSE +4 cm_ ± SD = 488 ± 41 ms) but a significant decrease in RTs when comparing PSE – 4 cm to PSE trials (t(29) = -6.0, p = 10-6 , d = -1.1; mean RT_PSE - 4 cm_ ± SD = 465 ± 47 ms) and PSE – 4 cm to PSE + 4 cm trials (t(29) = -5.5, p = 10-5 , d = -1.0; Figure S1A). However, no significant difference in uncertainty was found between PSE – 4 cm and PSE + 4 cm (percentage of left target choices in PSE + 4 cm [mean ± SD = 6.3 ± 5.3 %] and percentage of right target choices in PSE – 4 cm [mean ± SD = 4.6 ± 7.1 %]; t(29) = -1.3; p = 0.639, d = -0.23) but highly significant differences between the proportion of left target choices at PSE (least preferred target: mean ± SD = 41.4 ± 11.2 %) and the two other conditions (PSE – 4 cm vs PSE: t(29) = -13.0; p < 10-10 ; d = -2.4; PSE + 4 cm vs PSE: t(29) = -16.4; p < 10-10 ; d = -3.0; Figure S1B). Additionally, results from one-target trials suggest that target distance influenced RTs as they were significantly higher for movements toward the left target at PSE + 4 cm than at PSE – 4 cm (Bonferroni-corrected t-tests; t(29) = 4.2; p = 10-4; d = 0.77; mean RT_PSE + 4 cm_ = 385 ± 28 ms; mean RT_PSE – 4 cm_ = 375 ± 24 ms;) as well as when comparing RTs when the left target was located at PSE + 4 cm as compared to when it was at PSE (t(29) = 3.3; p = 0.009; d = 0.59; mean RT_PSE_ = 379 ± 28 ms), but not for PSE in comparison to PSE – 4 cm (t(29) = 2.1; p = 0.141; d = 0.38; Figure S1C).

As a consequence, if modulating decision uncertainty was truly inefficient in modifying RTs in our task, we should have seen no difference in RTs between the configurations in which the left target was located at PSE – 4 cm and the one at PSE. However, the pattern of behavioral results found is coherent with a co-influence of target distance and uncertainty on RTs (see Figure S1D below), therefore explaining the lack of significant difference in RTs between FreeEasy and FreeHard.

Figure S2


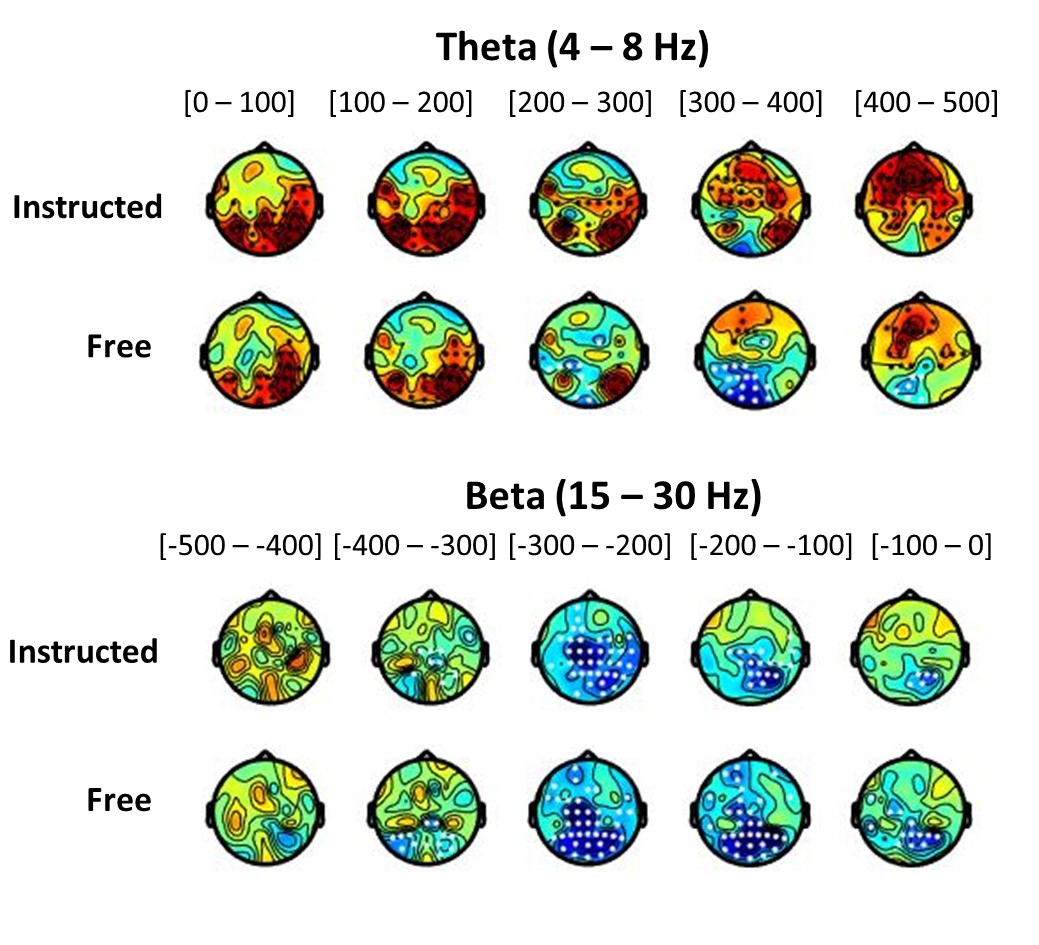


**Comparison of theta and beta power between two-target (Free and Instructed) and one-target trials (1T).** Results of the cluster-based permutation tests for each paired comparison. Each topographical plot represents the average difference in theta or beta power between the tested conditions for each electrode in 100-ms windows. Time windows are indicated above each topographical plot and reported in ms, according to stimulus onset for theta power, and movement onset for beta power. Hot colors indicate an increase and cold colors a decrease in power between the tested conditions. Black dots indicate electrodes belonging to a significant positive cluster (theta/beta increase) and white dots indicate electrodes belonging to a significant negative cluster (theta/beta decrease).

Detailed analysis:

The comparison of theta power between Instructed Hard and 1T revealed one significant large positive cluster (tsum = 5431.7, p = 0.001, time = 0 to 500 ms after stimulus onset) spanning occipito-parietal electrodes at early RT and midfrontal electrodes at late RT (Figure S2, first row). The comparison of Free Hard and 1T was qualitatively different, with an early increase in theta power at occipito-parietal electrodes in Free Hard (tsum = 1478.8, p = 0.001, time = 0 to 260 ms after stimulus onset), followed by a second moderate increase at midfrontal electrodes (tsum = 535.9, p = 0.018, time = 360 to 500 ms after stimulus onset) and a decrease at left occipito-parietal electrodes (tsum = -442.8, p = 0.027, time = 280 to 410 ms after stimulus onset) (Figure S2, second row). In contrast to the theta results, the modulations in beta power were qualitatively very similar across the two contexts. Indeed, there was a similar decrease in contralateral sensorimotor beta power when comparing Instructed Hard with 1T (tsum = -1408.5, p = 0.001, time = 300 to 40 ms before movement onset; Figure S2, third row) and Free Hard with 1T (tsum = -2122.4, p = 0.001, time = 340 to 40 ms before movement onset; Figure S2; fourth row).
